# Use of MALDI-TOF Mass Spectrometry for the Fast Identification of Gram-Positive Fish Pathogens

**DOI:** 10.3389/fmicb.2017.01492

**Published:** 2017-08-09

**Authors:** Gabriella B. N. Assis, Felipe L. Pereira, Alexandra U. Zegarra, Guilherme C. Tavares, Carlos A. Leal, Henrique C. P. Figueiredo

**Affiliations:** AQUACEN, National Reference Laboratory for Aquatic Animal Diseases, Ministry of Agriculture, Livestock and Food Supply, Federal University of Minas Gerais Belo Horizonte, Brazil

**Keywords:** MALDI-TOF MS, *S. agalactiae*, *S. iniae*, *S. dysgalactiae* subsp. *dysgalactiae*, *Lactococcus garvieae*

## Abstract

Gram-positive cocci, such as *Streptococcus agalactiae, Lactococcus garvieae, Streptococcus iniae*, and *Streptococcus dysgalactiae* subsp. *dysgalactiae*, are found throughout the world, particularly in outbreaks in farmed fish, and are thus associated with high economic losses, especially in the cultivation of Nile Tilapia. The aim of this study was to evaluate the efficacy of matrix-assisted laser desorption ionization (MALDI)-time of flight (TOF) mass spectrometry (MS) as an alternative for the diagnosis of these pathogens. One hundred and thirty-one isolates from Brazilian outbreaks assisted by the national authority were identified using a MALDI Biotyper from Bruker Daltonics. The results showed an agreement with respect to identification (Kappa = 1) between this technique and 16S ribosomal RNA gene sequencing for *S. agalactiae* and *L. garvieae*. However, for *S. iniae* and *S. dysgalactiae* subsp. *dysgalactiae*, perfect agreement was only achieved after the creation of a custom main spectra profile, as well as further comparisons with 16S ribosomal RNA and multilocus sequence analysis. MALDI-TOF MS was shown to be an efficient technology for the identification of these Gram-positive pathogens, yielding a quick and precise diagnosis.

## Introduction

Gram-positive cocci infections pose a great threat to farmed fish worldwide (Evans et al., 2002; Agnew and Barnes, 2007; Abdelsalam et al., 2013) and especially impact warm water systems used for the cultivation of Nile tilapia, one of the major commodities of global aquaculture (FAO, 2016). Four pathogens that are highly associated with outbreaks in fish farms are *Streptococcus agalactiae, Lactococcus garvieae, Streptococcus iniae*, and *S. dysgalactiae* subsp. *dysgalactiae* (SDD) (Evans et al., 2002; Agnew and Barnes, 2007; Mian et al., 2009; Netto et al., 2011; Figueiredo et al., 2012; Abdelsalam et al., 2013). *Streptococcus agalactiae, S. iniae*, and *L. garvieae* cause septicemia and meningoencephalitis in several species of marine and freshwater fish (Eldar et al., 1995; Evans et al., 2002; Mian et al., 2009; Figueiredo et al., 2012; Godoy et al., 2013; Soto et al., 2015; Fukushima et al., 2017). In fish, SDD infections are characterized by a systemic multifocal inflammatory reaction and a focal necrosis of the caudal peduncle, with moderate to high mortality rates during outbreaks (Nomoto et al., 2006).

Currently, the most widely used technology for the diagnosis of these infectious diseases is the isolation of the etiological agent in blood agar medium and subsequent identification through phenotypic/biochemical tests (Vendrell et al., 2006; Figueiredo et al., 2012; Assis et al., 2016). However, the performance of these tests can lead to misidentification or a lack of species-level resolution (Brigante et al., 2006; Tavares et al., 2016). Alternative molecular methods, such as species-specific PCR (Poyart et al., 1998) and the amplification and sequencing of the 16S ribosomal RNA (rRNA) gene, are useful for diagnosis (Kolbert and Persing, 1999; Patel, 2001; Clarridge, 2004) but are expensive and time consuming, mostly in trials with large number of clinical samples.

Recently, another technology to identify microorganisms was released: matrix-assisted laser desorption ionization (MALDI)-time of flight (TOF) mass spectrometry (MS) (Clark et al., 2013; Singhal et al., 2015). In this technique, the identification of the bacterial species is done by a comparison of peptide mass fingerprints to the device database. A typical mass range of 2–20 kDa is used, which represents mainly ribosomal proteins, along with a few housekeeping proteins (Singhal et al., 2015). There are many studies demonstrating the efficiency of MALDI-TOF MS in the classification of several species in a shorter time and with a lower cost (Bilecen et al., 2015), including typing (Nagy et al., 2011; Rizzardi et al., 2013) or identification of specific markers such as methicillin resistance (Østergaard et al., 2015; Ueda et al., 2016). Furthermore, MALDI-TOF MS can be performed in a short time for a wide range pathogens in one experiment (Bizzini and Greub, 2010). Additionally, it does not need a high level of staff training, reducing the risk of laboratory-associated infections by minimizing handling of living culture materials needed for the preparation of isolates.

Thus, the aim of this study was to evaluate the efficacy of MALDI-TOF MS for the identification of four Gram-positive cocci, *S. agalactiae, L. garvieae, S. iniae*, and SDD isolated from the kidneys, brains or abscesses of diseased fish from different geographic locations between 2003 and 2016.

## Materials and methods

### Bacterial strains

Bacterial strains were selected from the culture collection of the National Reference Laboratory for Aquatic Animal Diseases (AQUACEN) of the Brazilian Ministry of Agriculture, Livestock and Food Supply. These *S. agalactiae* (*n* = 50), *L. garvieae* (*n* = 11), *S. iniae* (*n* = 47), and SDD (*n* = 23) strains were isolated during bacteriological analyses of outbreaks in Brazilian fish farms in different years and geographical locations (Table S1). The isolation of these microorganisms was performed on chilled fish that were sent to AQUACEN for diagnosis. Swabs from brains, kidneys or abscesses were aseptically sampled and streaked onto 5% sheep blood agar (SBA) for the isolation of bacterial pathogens. These plates were incubated at 28°C for 48 h. Finally, the identification of bacterial species was carried out as previously described (Mian et al., 2009; Netto et al., 2011; Figueiredo et al., 2012; Fukushima et al., 2017).

### Species confirmation through 16S rRNA gene sequencing

The isolates were thawed and streaked onto 5% SBA and were incubated at 28°C for 48 h. Isolates were incubated in a lysozyme solution at 37°C overnight. Bacterial DNA was extracted with a Maxwell 16 Tissue DNA purification kit (Promega, Madison, WI, USA) according to the manufacturer's instructions. The extracted DNA was quantified using a Nanodrop spectrophotometer (Thermo Scientific, Wilmington, DE, USA). The purity of the extracted DNA was determined using the absorbance ratio at 260/280 nm. Samples with ratio of 1.8 ± 0.5 were stored at −80°C until use.

The 16S rRNA gene was amplified by PCR with the universal primers B37 (5′-TAC GGY TAC CTT GTT ACG A-3′) and C70 (5′-AGA GTT TGA TYM TGGC-3′) and PCR amplicons were purified according to the method described by Fox et al. (1995) for all strains used in this work. The sequencing reactions were performed using a BigDye™ Terminator Cycle Sequencing Kit (Applied Biosystems, UK) and evaluated with an ABI 3,500 Genetic Analyzer (Life Technologies, USA). Forward and reverse sequencing products were used to generate contigs with the BioEdit software (Ibis Biosciences, Carlsbad, USA) version 7.2. Their identity was evaluated using the BLAST webserver (http://www.ncbi.nlm.nih.gov/BLAST) by checking against existing sequences in the nt/nr database. A similarity of ≥ 97% was considered as the same species in accordance with Nguyen et al. (2016) and Větrovský and Baldrian (2013).

### MALDI-TOF MS real-time identification analysis

All isolates were thawed and streaked onto 5% SBA and incubated at 28°C for 48 h. A fresh, single colony of each bacterial strain was spotted using a toothpick into a target steel plate. For each strain, 1 μl of formic acid (70%) and 1 μl of MALDI-TOF MS matrix, consisting of a saturated solution of α-cyano-4-hydroxycinnamic acid (HCCA) (Bruker Daltonics, Bremen, Germany), were applied to the spot and allowed to air-dry. Spectra were acquired using the FlexControl MicroFlex LT mass spectrometer (Bruker Daltonics) with a 60-Hz nitrogen laser, in which up to 240 laser shots are fired in spiral movements to collect 40 shot steps for each strain spot. Furthermore, parameters for mass range detection were defined to allow the identification from 1,960 to 20,137 m/z, where Ion source 1 v was 19.99 kv, Ion source 2 voltage was 18.24 kv and the lens voltage was 6.0 kv for data acquisition. Prior to measurements, calibration was preceded with a bacterial test standard (*E. coli* DH5 alpha; Bruker Daltonics). The Real Time (RT) identification score criteria used were those recommended by the manufacturer: score ≥ 2.000 indicates a species-level identification, score ≥1.700 and <2.000 indicates a genus-level identification, and a score <1.700 indicates no reliable identification. Comparisons between MALDI-TOF MS strain identifications and those of other techniques were performed with R software version 3.0.1 (R Core Team, 2013) with the agreement rates determined by the Kappa coefficient.

### Creation of a custom main spectra profile

To identify possible *S. iniae* strains and to enhance the *S. dysgalactiae* discrimination at the subspecies-level in a MALDI Biotyper, Main Spectra Profiles (MSPs) were created with reference strains for each species. Fresh colonies of the *S. iniae* SI23 strain and the SDD SD64, SD92 and SD142 strains were extracted according Alatoom et al. (2011). Briefly, the strains were collected from the agar and added to 300 μl of distilled water, followed by the addition of 900 μl of ethanol. Two rounds of centrifugation for 2 min at 13,000 rpm and the complete removal of supernatant was necessary to obtain dried pellets. The pellets were suspended in 50 μl of formic acid (70%) and vortexed. Finally, 50 μl of acetonitrile was added and the mixtures were centrifuged for 2 min at 13,000 rpm. For assays, one microliter of the supernatant was spotted eight times onto a steel target. Directly after air-drying, each spot was overlaid with 1 μl of HCCA matrix. Each spot was measured three times with the same protocol/parameters described in the section above. The obtained spectra were closely analyzed in the FlexAnalysis software (Bruker Daltonics) to assess the high level of reproducibility. Finally, the spectra of each strain were uploaded to the MALDI Biotyper software version 3 (Bruker Daltonics) and assembled to generate a Main Spectra Profile (MSP) for the strains using the BioTyper MSP creation standard method. All steps were done according to the manufacturer's recommendations.

A figure illustrating the SD64 spectra was generated using R software version 3.0.1 (R Core Team, 2013), using data exported from the FlexAnalysis software (Bruker Daltonics). In addition, in order to compare the custom MSPs with the MSP preloaded on the Bruker MSP library, the BioTyper software version 3.0 (Bruker Daltonics) was used to perform a dendrogram analysis. The parameters used were distance measure = “correlation,” linkage = “average,” maximum number of top level nodes = “0,” score oriented dendrogram “enabled,” score threshold values for a single organism = “300,” and score threshold values for a related organism = “0.”

### *Streptococcus dysgalactiae* subspecies confirmation

The SDD strains that had subspecies suggested by Costa et al. (2014) were inferred by a BLAST comparison of the 16S rRNA and *sodA* genes, and the MALDI Biotyper (Bruker Daltonics) analysis suggested a closer relationship with *S. dysgalactiae* subsp. *equisimilis* (SDE). In addition to the 16S sequencing described above, a Next-Generation Sequence (NGS) experiment was performed. Three strains (SD64, SD92, and SD142) with different pulse-field gel electrophoresis (PFGE) profiles described in previous work from our group (Costa et al., 2014) were sequenced. DNA from the SDD strain was isolated from an overnight culture using a Maxwell 16 tissue DNA purification kit using the Maxwell 16 system (both from Promega). Sequencing was conducted on the Ion Torrent Personal Genome Machine sequencing system (Life Technologies) using a 200 bp fragment library kit, according to the manufacturer's recommendations. The barcodes of the raw data were removed using an in-house script (https://github.com/aquacen/fast_sample), and assembly was performed using SPAdes v3.9.1 (Nurk et al., 2013).

SDD taxonomic classification was determined using the Jensen and Kilian (2012) method, where the analysis of the phylogenetic relationship of seven housekeeping genes (*map, pfl, ppaC, pyk, rpoB, sodA*, and *tuf*) through a multilocus sequence analysis (MLSA) represent an improved basis for the identification of clinically important streptococci. The concatenated sequence of these housekeeping genes is used to establish differences between species that allow a more accurate identification within the pyogenic group of streptococci. The sequence of the draft genome of SDD ATCC 27957 is available on GenBank (Accession number: CM001076) and together with the genes of 30 streptococci strains submitted with the work of Jensen and Kilian (2012) were downloaded (Accession numbers: *map*: JN632385 to JN632479; *pfl*: JN632290 to JN632384; *ppaC*: JN632195 to JN632289; *pyk*: JN632100 to JN632194; *rpoB*: JN632005 to JN632099; *sodA*: JN631910 to JN632004; *tuf* : JN631815 to JN631909).

To extract the sequences of the corresponding housekeeping genes, a homology search for each of the seven genes in the SD64, SD92, and SD142 strains was performed using the BLAST webserver (http://www.ncbi.nlm.nih.gov/BLAST), with contigs generated by assembly software. The same strategy was performed with the SDD ATCC 27957 strain. All genes for each strain were concatenated in the following order: *map-pfI-ppaC-pyk-rpoB-sodA-tuf*. Alignment and phylogeny analyses were performed using MEGA6 (Tamura et al., 2013), with the Kimura-2 model parameters, using the Minimum Evolution algorithm, and a bootstrap of 1,000 replications.

## Results

### Species confirmation through 16S rRNA gene sequencing

The sequences of the 16S rRNA PCR products, which were generated with the aforementioned forward and reverse primers, were comprised in contigs for each strain. The mean lengths of the contigs were 1,514 ± 12, 1,537 ± 14, 1,519 ± 15, and 1,515 ± 17 bp for *S. agalactiae, L. garvieae, S. iniae*, and SDD, respectively. The contigs from each strain were used as queries for the BLAST webserver, and a percentage value of the similarities for *L. garvieae* was between 98 and 100, whereas *S. agalactiae, S. iniae* and SDD varied between 97 and 100. For the SDD strains, it was not possible make identification at the subspecies-level. For each SDD isolate there were results referring to the SDE and SDD with the same percentage value of identity that referred to the same query coverage.

### MALDI-TOF MS RT identification of *S. agalactiae* and *L. garvieae*

For each strain-spot, 1–3 spectra were expected, according to the manufacturer's instructions for quality assurance performed by MALDI Biotyper software of acquisition. For *S. agalactiae*, 64 spectra were acquired, whereas 11 spectra were acquired for *L. garvieae*. All strains for both species were identified at the species-level (score ≥ 2.000). The minimal and maximal scores for *S. agalactiae* were 2.083 and 2.377 (Table 1), respectively, and for *L. garvieae* were 2.081 and 2.218 (Table 2), respectively. For both species a perfect agreement (Kappa = 1; CI: 1.0–1.0; and *p* < 0.005) was observed between the 16S rRNA gene sequencing and MALDI-TOF MS techniques to identify the species.

**Table 1:** *Streptococcus agalactiae* strains identification by 16S rRNA sequencing and MALDI-TOF MS.

**Strain**	**16S rRNA sequencing**		**MALDI Biotyper**	
	**Species**	**% Identity**	**Organism best match**	**Score value**
SA001	*Streptococcus agalactiae*	100	*Streptococcus agalactiae*	2.330
SA005	*Streptococcus agalactiae*	100	*Streptococcus agalactiae*	2.318
SA007	*Streptococcus agalactiae*	100	*Streptococcus agalactiae*	2.371
SA009	*Streptococcus agalactiae*	100	*Streptococcus agalactiae*	2.302
SA016	*Streptococcus agalactiae*	100	*Streptococcus agalactiae*	2.357
SA020	*Streptococcus agalactiae*	100	*Streptococcus agalactiae*	2.296
SA030	*Streptococcus agalactiae*	100	*Streptococcus agalactiae*	2.289
SA033	*Streptococcus agalactiae*	100	*Streptococcus agalactiae*	2.211
SA053	*Streptococcus agalactiae*	100	*Streptococcus agalactiae*	2.206
SA073	*Streptococcus agalactiae*	100	*Streptococcus agalactiae*	2.259
SA075	*Streptococcus agalactiae*	100	*Streptococcus agalactiae*	2.189
SA079	*Streptococcus agalactiae*	100	*Streptococcus agalactiae*	2.251
SA081	*Streptococcus agalactiae*	100	*Streptococcus agalactiae*	2.327
SA085	*Streptococcus agalactiae*	100	*Streptococcus agalactiae*	2.207
SA095	*Streptococcus agalactiae*	100	*Streptococcus agalactiae*	2.275
SA097	*Streptococcus agalactiae*	100	*Streptococcus agalactiae*	2.227
SA102	*Streptococcus agalactiae*	99	*Streptococcus agalactiae*	2.172
SA117	*Streptococcus agalactiae*	97	*Streptococcus agalactiae*	2.162
SA132	*Streptococcus agalactiae*	100	*Streptococcus agalactiae*	2.322
SA136	*Streptococcus agalactiae*	100	*Streptococcus agalactiae*	2.220
SA159	*Streptococcus agalactiae*	100	*Streptococcus agalactiae*	2.364
SA172	*Streptococcus agalactiae*	100	*Streptococcus agalactiae*	2.339
SA184	*Streptococcus agalactiae*	100	*Streptococcus agalactiae*	2.306
SA191	*Streptococcus agalactiae*	100	*Streptococcus agalactiae*	2.207
SA201	*Streptococcus agalactiae*	100	*Streptococcus agalactiae*	2.221
SA209	*Streptococcus agalactiae*	100	*Streptococcus agalactiae*	2.309
SA212	*Streptococcus agalactiae*	100	*Streptococcus agalactiae*	2.377
SA218	*Streptococcus agalactiae*	100	*Streptococcus agalactiae*	2.331
SA220	*Streptococcus agalactiae*	100	*Streptococcus agalactiae*	2.351
SA245	*Streptococcus agalactiae*	100	*Streptococcus agalactiae*	2.192
SA256	*Streptococcus agalactiae*	100	*Streptococcus agalactiae*	2.083
SA289	*Streptococcus agalactiae*	100	*Streptococcus agalactiae*	2.167
SA330	*Streptococcus agalactiae*	100	*Streptococcus agalactiae*	2.317
SA333	*Streptococcus agalactiae*	100	*Streptococcus agalactiae*	2.294
SA341	*Streptococcus agalactiae*	100	*Streptococcus agalactiae*	2.296
SA343	*Streptococcus agalactiae*	100	*Streptococcus agalactiae*	2.276
SA346	*Streptococcus agalactiae*	100	*Streptococcus agalactiae*	2.254
SA374	*Streptococcus agalactiae*	100	*Streptococcus agalactiae*	2.363
SA375	*Streptococcus agalactiae*	100	*Streptococcus agalactiae*	2.360
SA623	*Streptococcus agalactiae*	100	*Streptococcus agalactiae*	2.248
SA627	*Streptococcus agalactiae*	100	*Streptococcus agalactiae*	2.349
SA665	*Streptococcus agalactiae*	97	*Streptococcus agalactiae*	2.281
SA719	*Streptococcus agalactiae*	98	*Streptococcus agalactiae*	2.197
SA796	*Streptococcus agalactiae*	97	*Streptococcus agalactiae*	2.359
SA808	*Streptococcus agalactiae*	97	*Streptococcus agalactiae*	2.242
SA887	*Streptococcus agalactiae*	97	*Streptococcus agalactiae*	2.185
SA929	*Streptococcus agalactiae*	99	*Streptococcus agalactiae*	2.230
SA941	*Streptococcus agalactiae*	97	*Streptococcus agalactiae*	2.257
SA959	*Streptococcus agalactiae*	97	*Streptococcus agalactiae*	2.328
SA972	*Streptococcus agalactiae*	97	*Streptococcus agalactiae*	2.183

**Table 2:** *Lactococcus garvieae* strains identification by 16S rRNA sequencing and MALDI-TOF MS.

**Strain**	**16S rRNA sequencing**		**MALDI Biotyper**	
	**Species**	**% Identity**	**Organism best match**	**Score value**
LG002	*Lactococcus garvieae*	100	*Lactococcus garvieae*	2.166
LG005	*Lactococcus garvieae*	99	*Lactococcus garvieae*	2.195
LG009	*Lactococcus garvieae*	98	*Lactococcus garvieae*	2.084
LG010	*Lactococcus garvieae*	98	*Lactococcus garvieae*	2.218
LG011	*Lactococcus garvieae*	100	*Lactococcus garvieae*	2.213
LG015	*Lactococcus garvieae*	100	*Lactococcus garvieae*	2.142
LG018	*Lactococcus garvieae*	99	*Lactococcus garvieae*	2.110
LG019	*Lactococcus garvieae*	98	*Lactococcus garvieae*	2.114
LG020	*Lactococcus garvieae*	100	*Lactococcus garvieae*	2.184
LG021	*Lactococcus garvieae*	99	*Lactococcus garvieae*	2.165
LG022	*Lactococcus garvieae*	98	*Lactococcus garvieae*	2.081

### MALDI-TOF MS RT identification of *S. iniae*

A total of 52 spectra were obtained for the 47 strains. Identification of *S. iniae* was possible in ~53% of isolates at the genus-level (Table 3), and the minimal and maximal scores were 1.482 and 1.854, respectively, including 22 with no reliable identification. The genus-level was inferred by an approximation of the spectra with *S. dysgalactiae* (*n* = 7), *S. equi* (*n* = 1), and *S. pyogenes* (*n* = 17). The species identification agreement when comparing 16S rRNA gene sequencing and MALDI-TOF MS was poor (Kappa = 0.04; CI: −0.03 to 0.11; and *p* = 0.063).

**Table 3:** *Streptococcus iniae* strains identification by 16S rRNA sequencing and MALDI-TOF MS (before and after custom MSP inclusion).

**Strain**	**16S rRNA sequencing**		**MALDI Biotyper**			
			**Before custom MSP inclusion**		**After custom MSP inclusion**	
	**Species**	**% Identity**	**Organism best match**	**Score value**	**Organism best match**	**Score value**
SI022	*Streptococcus iniae*	98	*Streptococcus pyogenes*	1.736^a^	*S. iniae* SI23	2.223
SI023	*Streptococcus iniae*	99	Not reliable identification	1.509	*S. iniae* SI23	2.089
SI024	*Streptococcus iniae*	99	*Streptococcus dysgalactiae*	1.741^a^	*S. iniae* SI23	2.165
SI025	*Streptococcus iniae*	100	Not reliable identification	1.580	*S. iniae* SI23	2.205
SI027	*Streptococcus iniae*	100	Not reliable identification	1.642	*S. iniae* SI23	2.148
SI028	*Streptococcus iniae*	100	Not reliable identification	1.683	*S. iniae* SI23	2.013
SI029	*Streptococcus iniae*	97	*Streptococcus pyogenes*	1.724^a^	*S. iniae* SI23	2.199
SI444	*Streptococcus iniae*	99	Not reliable identification	1.627	*S. iniae* SI23	2.031
SI503	*Streptococcus iniae*	97	*Streptococcus dysgalactiae*	1.737^a^	*S. iniae* SI23	2.301
SI674	*Streptococcus iniae*	98	Not reliable identification	1.664	*S. iniae* SI23	2.205
SI677	*Streptococcus iniae*	99	*Streptococcus pyogenes*	1.732^a^	*S. iniae* SI23	2.409
SI692	*Streptococcus iniae*	99	*Streptococcus equi*	1.700^a^	*S. iniae* SI23	2.332
SI696	*Streptococcus iniae*	99	Not reliable identification	1.620	*S. iniae* SI23	2.308
SI698	*Streptococcus iniae*	97	Not reliable identification	1.679	*S. iniae* SI23	2.426
SI699	*Streptococcus iniae*	98	*Streptococcus dysgalactiae*	1.821^a^	*S. iniae* SI23	2.273
SI700	*Streptococcus iniae*	98	Not reliable identification	1.605	*S. iniae* SI23	2.147
SI701	*Streptococcus iniae*	97	Not reliable identification	1.629	*S. iniae* SI23	2.272
SI702	*Streptococcus iniae*	97	Not reliable identification	1.675	*S. iniae* SI23	2.281
SI705	*Streptococcus iniae*	98	Not reliable identification	1.678	*S. iniae* SI23	2.326
SI706	*Streptococcus iniae*	99	*Streptococcus pyogenes*	1.750^a^	*S. iniae* SI23	2.122
SI711	*Streptococcus iniae*	99	*Streptococcus pyogenes*	1.733^a^	*S. iniae* SI23	2.231
SI712	*Streptococcus iniae*	97	*Streptococcus pyogenes*	1.749^a^	*S. iniae* SI23	2.124
SI713	*Streptococcus iniae*	98	*Streptococcus pyogenes*	1.713^a^	*S. iniae* SI23	2.275
SI714	*Streptococcus iniae*	99	Not reliable identification	1.641	*S. iniae* SI23	2.075
SI715	*Streptococcus iniae*	98	*Streptococcus pyogenes*	1.748^a^	*S. iniae* SI23	2.216
SI717	*Streptococcus iniae*	97	Not reliable identification	1.648	*S. iniae* SI23	2.261
SI718	*Streptococcus iniae*	98	*Streptococcus pyogenes*	1.825^a^	*S. iniae* SI23	2.249
SI720	*Streptococcus iniae*	98	*Streptococcus pyogenes*	1.829^a^	*S. iniae* SI23	2.321
SI790	*Streptococcus iniae*	97	*Streptococcus pyogenes*	1.774^a^	*S. iniae* SI23	2.204
SI791	*Streptococcus iniae*	97	*Streptococcus dysgalactiae*	1.781^a^	*S. iniae* SI23	2.255
SI792	*Streptococcus iniae*	99	Not reliable identification	1.556	*S. iniae* SI23	2.054
SI797	*Streptococcus iniae*	98	*Streptococcus pyogenes*	1.854^a^	*S. iniae* SI23	2.043
SI798	*Streptococcus iniae*	97	Not reliable identification	1.675	*S. iniae* SI23	2.293
SI819	*Streptococcus iniae*	97	*Streptococcus pyogenes*	1.787^a^	*S. iniae* SI23	2.203
SI826	*Streptococcus iniae*	98	*Streptococcus pyogenes*	1.809^a^	*S. iniae* SI23	2.244
SI831	*Streptococcus iniae*	98	Not reliable identification	1.557	*S. iniae* SI23	2.313
SI839	*Streptococcus iniae*	97	*Streptococcus dysgalactiae*	1.738^a^	*S. iniae* SI23	2.173
SI841	*Streptococcus iniae*	99	Not reliable identification	1.686	*S. iniae* SI23	2.242
SI842	*Streptococcus iniae*	97	Not reliable identification	1.614	*S. iniae* SI23	2.379
SI852	*Streptococcus iniae*	97	*Streptococcus dysgalactiae*	1.707^a^	*S. iniae* SI23	2.071
SI870	*Streptococcus iniae*	97	Not reliable identification	1.482	*S. iniae* SI23	2.228
SI875	*Streptococcus iniae*	99	Not reliable identification	1.668	*S. iniae* SI23	2.182
SI876	*Streptococcus iniae*	99	*Streptococcus dysgalactiae*	1.751^a^	*S. iniae* SI23	2.238
SI913	*Streptococcus iniae*	98	Not reliable identification	1.625	*S. iniae* SI23	2.276
SI928	*Streptococcus iniae*	99	*Streptococcus pyogenes*	1.819^a^	*S. iniae* SI23	2.031
SI954	*Streptococcus iniae*	99	*Streptococcus pyogenes*	1.802^a^	*S. iniae* SI23	2.214
SI970	*Streptococcus iniae*	99	*Streptococcus pyogenes*	1.853^a^	*S. iniae* SI23	2.241

a *Genus-level identification*.

**Figure 1:**
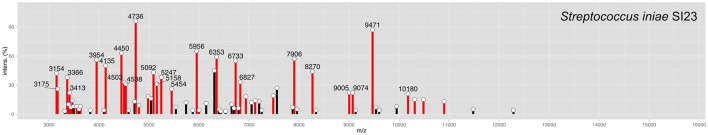
MSP of *S. iniae* SI23 peak identification. Peaks with intensities greater than 20% are labeled. Peaks with a previously identified m/z (Kim et al., 2017) are shown in red bars.

**Figure 2:**
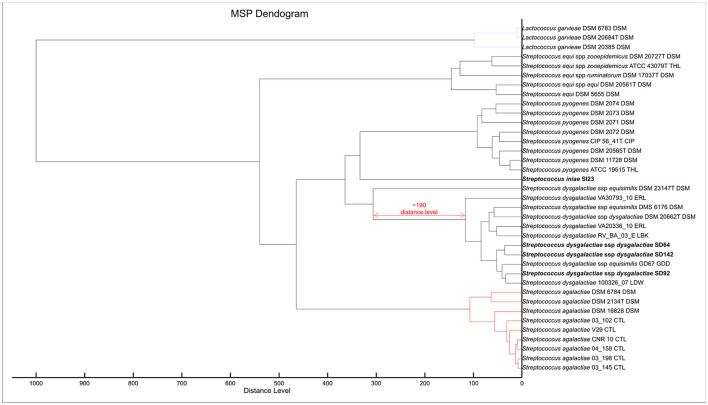
MSP Dendrogram analysis of the custom MSP and Bruker MSP library of *Lactococcus garvieae* and *Streptococcus* spp. Isolates from this work are in bold. *S. iniae* SI23 is alone and between the *S. pyogenes* and *S. dysgalactiae* spp. clades. SDD is in an intra-species-specific clade of *S. dysgalactiae* spp. strains. The red arrow shows a distance level of ~190 from *S. dysgalactiae* spp. *equisimilis* DSM 23147^T^ to other *S. dysgalactiae* strains.

To make possible the correct identification of *S. iniae* strains using the MALDI Biotyper, a custom MSP was created for this species (Figure 1; MSP available at http://www.renaqua.gov.br/aquacen-msp-si/). Twenty-four spectra were collected for one isolate (SI23) by the Biotyper RTC program. The spectra were analyzed in the FlexAnalysis software to identify a high level of reproducibility, and all spectra were used to create the MSP. A dendrogram generated in BioTyper software (Figure 2) shows the SI23 strain as a single leaf between the *S. pyogenes* and *S. dysgalactiae* clades. After the inclusion of the custom MSP of *S. iniae*, all the strains were identified at the species-level (Table 3), and the minimal and maximal score values were 2.013 and 2.426, respectively. A complete agreement between both tested techniques was observed (Kappa = 1; CI: 1.0–1.0; and *p* < 0.005) for species identification.

### MALDI-TOF MS RT identification of SDD

The identification of SDD isolates, using 25 spectra from 23 strains, was obtained by an approximation of *S. dysgalactiae* and SDE MSPs at the species-level. Minimal and maximal scores were 2.058 and 2.298, respectively. Of all the SDD strains, 13 were identified with proximity to the subspecies *equisimilis*, and in 10 strains, there was no discrimination of subspecies (Table 4). The agreement between techniques was perfect when considering the species-level (Kappa = 1; CI: 1.0–1.0; and *p* < 0.004), but when considering the subspecies-level the agreement was only fair (Kappa = 0.21; CI: −0.08−0.52; *p* = 0.075). This demonstrated that both techniques were unable to identify strains at the subspecies-level.

**Table 4:** SDD strains identification by 16S rRNA sequencing and MALDI-TOF MS (before and after custom MSP inclusion).

**Strain**	**16S rRNA sequencing**		**MALDI Biotyper**			
			**Before custom MSP inclusion**		**After custom MSP inclusion**	
	**Species**	**% Identity**	**Organism best match**	**Score value**	**Organism best match**	**Score value**
SD054	*Streptococcus dysgalactiae*	97	*Streptococcus dysgalactiae* ssp. *equisimilis*	2.116	*Streptococcus dysgalactiae* subsp. *dysgalactiae* SD64	2.497
SD056	*Streptococcus dysgalactiae*	97	*Streptococcus dysgalactiae* ssp. *equisimilis*	2.298	*Streptococcus dysgalactiae* subsp. *dysgalactiae* SD64	2.480
SD061	*Streptococcus dysgalactiae*	98	*Streptococcus dysgalactiae* ssp. *equisimilis*	2.251	*Streptococcus dysgalactiae* subsp. *dysgalactiae* SD64	2.438
SD064	*Streptococcus dysgalactiae*	100	*Streptococcus dysgalactiae* ssp. *equisimilis*	2.161	*Streptococcus dysgalactiae* subsp. *dysgalactiae* SD64	2.458
SD068	*Streptococcus dysgalactiae*	98	*Streptococcus dysgalactiae* ssp. *equisimilis*	2.130	*Streptococcus dysgalactiae* subsp. *dysgalactiae* SD142	2.497
SD092	*Streptococcus dysgalactiae*	100	*Streptococcus dysgalactiae* ssp. *equisimilis*	2.058	*Streptococcus dysgalactiae* subsp. *dysgalactiae* SD64	2.320
SD120	*Streptococcus dysgalactiae*	97	*Streptococcus dysgalactiae* ssp. *equisimilis*	2.151	*Streptococcus dysgalactiae* subsp. *dysgalactiae* SD64	2.346
SD137	*Streptococcus dysgalactiae*	98	*Streptococcus dysgalactiae*	2.122	*Streptococcus dysgalactiae* subsp. *dysgalactiae* SD142	2.338
SD140	*Streptococcus dysgalactiae*	97	*Streptococcus dysgalactiae* ssp. *equisimilis*	2.130	*Streptococcus dysgalactiae* subsp. *dysgalactiae* SD142	2.531
SD142	*Streptococcus dysgalactiae*	99	*Streptococcus dysgalactiae*	2.078	*Streptococcus dysgalactiae* subsp. *dysgalactiae* SD142	2.384
SD143	*Streptococcus dysgalactiae*	98	*Streptococcus dysgalactiae*	2.164	*Streptococcus dysgalactiae* subsp. *dysgalactiae* SD64	2.479
SD145	*Streptococcus dysgalactiae*	97	*Streptococcus dysgalactiae* ssp. *equisimilis*	2.175	*Streptococcus dysgalactiae* subsp. *dysgalactiae* SD142	2.548
SD280	*Streptococcus dysgalactiae*	97	*Streptococcus dysgalactiae*	2.165	*Streptococcus dysgalactiae* subsp. *dysgalactiae* SD142	2.471
SD281	*Streptococcus dysgalactiae*	97	*Streptococcus dysgalactiae*	2.192	*Streptococcus dysgalactiae* subsp. *dysgalactiae* SD64	2.277
SD282	*Streptococcus dysgalactiae*	98	*Streptococcus dysgalactiae*	2.071	*Streptococcus dysgalactiae* subsp. *dysgalactiae* SD142	2.511
SD283	*Streptococcus dysgalactiae*	97	*Streptococcus dysgalactiae* ssp. *equisimilis*	2.201	*Streptococcus dysgalactiae* subsp. *dysgalactiae* SD142	2.513
SD284	*Streptococcus dysgalactiae*	98	*Streptococcus dysgalactiae*	2.195	*Streptococcus dysgalactiae* subsp. *dysgalactiae* SD142	2.302
SD285	*Streptococcus dysgalactiae*	97	*Streptococcus dysgalactiae*	2.073	*Streptococcus dysgalactiae* subsp. *dysgalactiae* SD142	2.523
SD286	*Streptococcus dysgalactiae*	97	*Streptococcus dysgalactiae* ssp. *equisimilis*	2.180	*Streptococcus dysgalactiae* subsp. *dysgalactiae* SD142	2.461
SD287	*Streptococcus dysgalactiae*	99	*Streptococcus dysgalactiae* ssp. *equisimilis*	2.168	*Streptococcus dysgalactiae* subsp. *dysgalactiae* SD142	2.579
SD367	*Streptococcus dysgalactiae*	97	*Streptococcus dysgalactiae*	2.177	*Streptococcus dysgalactiae* subsp. *dysgalactiae* SD142	2.432
SD370	*Streptococcus dysgalactiae*	99	*Streptococcus dysgalactiae*	2.171	*Streptococcus dysgalactiae* subsp. *dysgalactiae* SD142	2.565
SD372	*Streptococcus dysgalactiae*	98	*Streptococcus dysgalactiae* ssp. *equisimilis*	2.143	*Streptococcus dysgalactiae* subsp. *dysgalactiae* SD64	2.366

**Figure 3:**
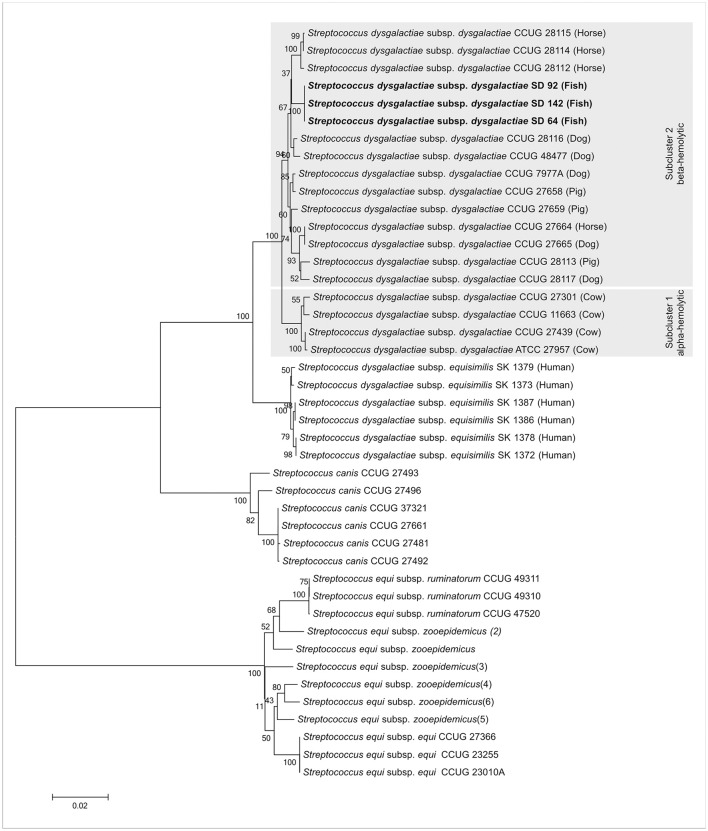
Tree taxonomy analysis of SDD SD64, SD92, and SD142 strains. The strains from this work (bold) form a specific clade with other SDD strains from Jensen and Kilian (2012). Gray hatched areas are the subcluster of alpha- and beta-hemolytic strains proposed by Jensen and Kilian (2012).

These strains, according to previous work of our group (Costa et al., 2014), are from SDD subspecies. Therefore, an NGS experiment was done to confirm the subspecies assignments. Contigs from the assembly of the strains SD64, SD92, and SD142 (data not shown) were used for a MLSA analysis. The three strains formed a clade with SDD from work of Jensen and Kilian (2012), confirming the classification of theses strains as SDD subspecies in accordance with the methodology used (Figure 3).

**Figure 4:**
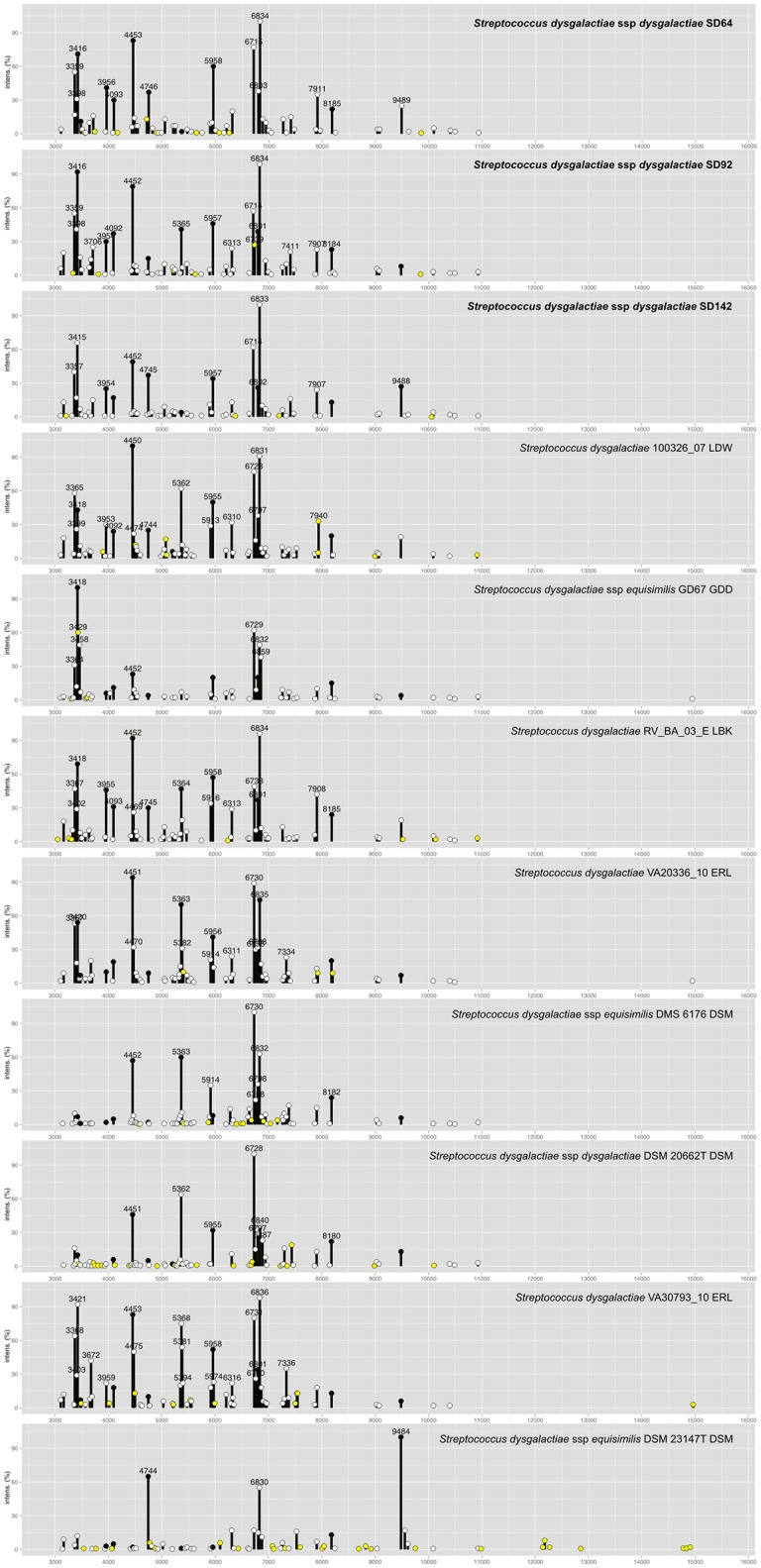
Main Spectra Profiles of *S. dysgalactiae* subsp. *dysgalactiae* SD64, SD92, and SD142 peaks identification. The strains of this work (bold) together with the *S. dysgalactiae* group from Bruker MSP library. Peaks with intensities greater than 20% are labeled. Peaks common of all MSP are plotted in black circles. Peaks common to two or more MSPs are plotted in white circles. Peaks exclusive of each MSP are plotted in yellow circles.

To improve the identification by the MALDI Biotyper, custom MSPs were created for SDD (Figure 4; MSP available at http://www.renaqua.gov.br/aquacen-msp-sdd/). Twenty-four spectra were collected for each isolate as described above and the spectra were analyzed in the FlexAnalysis, where all spectra were used to create the MSP. A dendrogram generated in BioTyper software (Figure 2) shows the SD64, SD92, and SD142 strains in an intra-species-specific clade of *S. dysgalactiae* spp. Figure 4 shows the common and exclusive peaks of custom MSPs and Bruker library MSPs, and, interestingly, the SDE DSM 23147^T^ shows 25 exclusive peaks.

After this inclusion (Table 4), all isolates matched with to the three included custom MSP for the three best matches (Table S2), with minimal and maximal scores of 2.277 and 2.579, respectively. The agreement between 16S rRNA gene sequencing and MALDI-TOF MS was poor (Kappa = 0.08, CI: −0.05−0.22; *p* = 0.050), considering that 16S rRNA gene sequencing was unable to identify subspecies, whereas with MALDI-TOF MS, they could be determined effectively. In contrast, considering the MLSA analysis, the agreement between this technique and MALDI-TOF MS was perfect (Kappa = 1; CI: 1.0–1.0; and *p* < 0.005).

## Discussion

Gram-positive cocci have been associated with acute and chronic fish diseases. They have become an increasingly important problem in the aquaculture industry in many countries (Evans et al., 2002; Vendrell et al., 2006; Agnew and Barnes, 2007; Mian et al., 2009; Netto et al., 2011; Figueiredo et al., 2012; Abdelsalam et al., 2013; Costa et al., 2014). An barrier to the better utilization of fish produced are the infectious diseases, including the control of the potential zoonotic infections caused by *S. iniae* (Keirstead et al., 2014). Thus, accelerating the diagnosis of diseases remains a big challenge. An alternative for these diagnoses is species-specific PCR and 16S rRNA gene sequencing, but these techniques are expensive, time consuming and require highly technical skills. Meanwhile, the MALDI-TOF MS method can be an important technique to increase the laboratory speeds of identification of the etiological agent because it is an efficient and cost-effective method for the rapid and routine identification of bacterial isolates in the clinical microbiology laboratory (Seng et al., 2009; Seibold et al., 2010). The potential for identification at the serotype or strain level, and antibiotic resistance profiling within minutes, makes MALDI-TOF MS an on-going revolution in the clinical microbiology laboratory (Romero-Gómez et al., 2012; Østergaard et al., 2015; Sauget et al., 2016; Ueda et al., 2016).

*Streptococcus agalactiae* and *Lactococcus garvieae* strains were classified as the correct species in 100% of the MALDI Biotyper experiments. Both species had been cited in previous works with MALDI-TOF MS systems (Lartigue et al., 2009; Navas et al., 2013), but not with regards to strains isolated from fish. Although there are no studies about the variation of the subtype of *L. garvieae*, a large number of *S. agalactiae* subtypes are known (Jones et al., 2003). The strains obtained from fish farm outbreaks in Brazil, used in this work, are from different genomic subtypes (Godoy et al., 2013), but nevertheless they did not show divergence in RT identification using the MALDI Biotyper.

The possibility of inclusion of a custom MSP on the Bruker MALDI Biotyper makes the tool expansive and allows for its adaptation to the laboratory business independent of the equipment manufacturer. Following the example of what had previously been reported by Segawa et al. (2015), the *S. iniae* SI23 strain and SDD SD64, SD92, and SD142 strains were included as MSPs, and the results improved to 100% correct identification. Recently, Fan et al. (2017), analyzing studies performed of streptococci rapid classification, suggested an overestimated accuracy of MALDI-TOF MS systems on *Streptococcus* spp. identification, since the 16S rRNA gene sequencing analyses were only performed on discrepant results. In our analysis, all strains were identified by 16S rRNA gene sequencing or by the 16S rRNA gene in addition to housekeeping genes that were sequenced in parallel with the MALDI-TOF MS experiments, in order to achieve more confident results.

*Streptococcus iniae* strains, before the inclusion of a custom MSP, had matches with *S. pyogenes* and *S. dysgalactiae*, with scores lower than 2.000, suggesting a genus-level match (Table 3) within only ~53% of tested isolates. The Bruker MSP library does not give MSP information about this species. The included custom MSP of SI23 showed similarities with these two species (Figure 2). These data corroborate with recent work from Kim et al. (2017) that shows the inclusion of *S. iniae* MSPs for the classification of *S. iniae* at the species-level, and shows the peaks list shared by *S. iniae* and *S. pyogenes*. Furthermore, 24 of 26 (~92%) of peaks with relative intensities greater than 20 are shared between *S. iniae* ATCC 29178 (Kim et al., 2017) and *S. iniae* SI23 (Figure 1).

In relation to the SDD strains, during the strains' RT classification, the results were all above 2.000; however, 13 strains were classified as SDE, and the other 10 were classified as *S. dysgalactiae* species (Table 4). In previous work from our group (Costa et al., 2014), we suggested that the Brazilian *S. dysgalactiae* isolates were from a *dysgalactiae* subspecies, according to 16S rRNA and *sodA* genes sequencing. Because of previous work (Jensen and Kilian, 2012) based on the MLSA analysis of a combination of seven housekeeping genes and the study of their phylogenetic relationships, an identification of the tested isolates in this work as SDD was confirmed. A custom MSP was created with the chosen isolates SD64, SD92, and SD142. Each strain has a different genotype that was identified in analyses made by PFGE in a previous work from our group (Costa et al., 2014). Using the custom MSP, all the analyzed strains had a correspondence larger than 2.000 (Table 4), indicating a high similarity of these strains with the created MSPs. Specimens in the Bruker MSP library named SDE and *S. dysgalactiae* do not have an accessible history, and the strain identified as SDD is referenced as ATCC® 43078™, which is an isolate from a cow with mastitis (Garvie et al., 1983). Furthermore, as Figure 2 shows, the SDE DSM 23147^*T*^ showed a distance level (i.e., similarity of selected isolates with a maximal value of divergence of 1,000) of ~190 from another clade of *S. dysgalactiae* isolates and a different partner using MSP profiles in Figure 4. This characteristic suggests, taking into consideration there is no traceable information for the isolates in addition to the recent studies of *S. dysgalactiae* spp. (Jensen and Kilian, 2012; Ciszewski et al., 2016), that a reclassification, based on genomic analyses, should be done for such isolates from the Bruker MSP library.

Although the MALDI Biotyper is primarily designed for diagnoses at the species-level, in our experiments it was possible to correctly identify the subspecies of SDD, allowing for a rapid and low cost analysis when compared with other techniques to make subspecies-level identifications. MALDI-TOF MS was shown to be an efficient technology for identifying important Gram-positive cocci that cause major diseases in farmed fish.

## Author contributions

GA, FP, and AZ wrote the manuscript. HF, GA, FP, GT, and CL conceived and designed the experiments. FP and AZ perform bioinformatics analyses. HF coordinated all analyses of the project. All authors read and approved the final manuscript.

### Conflict of interest statement

The authors declare that the research was conducted in the absence of any commercial or financial relationships that could be construed as a potential conflict of interest.
