# Supplementary material for: Use of MALDI-TOF Mass Spectrometry for the Fast Identification of Gram-Positive Fish Pathogens
Source: Front Microbiol. 2017 Aug 9;8:1492. doi: 10.3389/fmicb.2017.01492 (PMC5552964; doi:10.3389/fmicb.2017.01492)
Supplement: Supplementary file 1 [file Table1.DOCX]

Table S1. Strain history.

| **Strain** | **Year of isolation** | **Host isolation** | **Farm number** | **State** | **Organ** |
| --- | --- | --- | --- | --- | --- |
| LG002 | 2013 | *Oreochromis niloticus* | FARM #1 | MG | Brain |
| LG005 | 2013 | *Oreochromis niloticus* | FARM #1 | MG | Kidney |
| LG009 | 2013 | *Oreochromis niloticus* | FARM #1 | MG | Kidney |
| LG010 | 2014 | *Lophiosilurus alexandri* | FARM #1 | MG | Brain |
| LG011 | 2014 | *Oreochromis niloticus* | FARM #2 | MG | Kidney |
| LG015 | 2015 | *Oreochromis niloticus* | FARM #3 | SP | Brain |
| LG018 | 2016 | *Pseudoplatystoma corruscans* x *P. fasciatum* | FARM #4 | MS | Brain |
| LG019 | 2016 | *Pseudoplatystoma corruscans* x *P. fasciatum* | FARM #5 | MS | Brain |
| LG020 | 2016 | *Pseudoplatystoma corruscans* x *P. fasciatum* | FARM #5 | MS | Brain |
| LG021 | 2016 | *Pseudoplatystoma corruscans* x *P. fasciatum* | FARM #5 | MS | Brain |
| LG022 | 2016 | *Pseudoplatystoma corruscans* x *P. fasciatum* | FARM #5 | MS | Brain |
| SA001 | 2003 | *Oreochromis niloticus* | FARM #5 | MG | Brain |
| SA005 | 2004 | *Oreochromis niloticus* | FARM #6 | ES | Brain |
| SA007 | 2005 | *Oreochromis niloticus* | FARM #7 | BA | Kidney |
| SA009 | 2005 | *Oreochromis niloticus* | FARM #8 | ES | Brain |
| SA016 | 2006 | *Oreochromis niloticus* | FARM #9 | SP | Brain |
| SA020 | 2006 | *Oreochromis niloticus* | FARM #10 | PR | Kidney |
| SA030 | 2006 | *Oreochromis niloticus* | FARM #10 | SP | Brain |
| SA033 | 2006 | *Oreochromis niloticus* | FARM #11 | SP | Brain |
| SA053 | 2007 | *Oreochromis niloticus* | FARM #12 | CE | Brain |
| SA073 | 2008 | *Oreochromis niloticus* | FARM #13 | CE | Brain |
| SA075 | 2008 | *Oreochromis niloticus* | FARM #13 | CE | Brain |
| SA079 | 2009 | *Oreochromis niloticus* | FARM #13 | SC | Kidney |
| SA081 | 2009 | *Pseudoplatystoma corruscans* x *P. fasciatum* | FARM #13 | MT | Brain |
| SA085 | 2010 | *Oreochromis niloticus* | FARM #13 | Al | Brain |
| SA095 | 2010 | *Oreochromis niloticus* | FARM #13 | Al | Brain |
| SA097 | 2010 | *Oreochromis niloticus* | FARM #14 | PE | Brain |
| SA102 | 2010 | *Oreochromis niloticus* | FARM #15 | PE | Brain |
| SA117 | 2009 | *Pseudoplatystoma corruscans* x *P. fasciatum* | FARM #15 | MT | Brain |
| SA132 | 2011 | *Oreochromis niloticus* | FARM #15 | CE | Brain |
| SA136 | 2011 | *Oreochromis niloticus* | FARM #15 | CE | Brain |
| SA159 | 2011 | *Oreochromis niloticus* | FARM #15 | MG | Brain |
| SA172 | 2011 | *Oreochromis niloticus* | FARM #15 | ES | Brain |
| SA184 | 2011 | *Oreochromis niloticus* | FARM #16 | ES | Brain |
| SA191 | 2012 | *Oreochromis niloticus* | FARM #17 | CE | Brain |
| SA201 | 2012 | *Oreochromis niloticus* | FARM #18 | ES | Brain |
| SA209 | 2012 | *Oreochromis niloticus* | FARM #18 | SP | Brain |
| SA212 | 2012 | *Oreochromis niloticus* | FARM #18 | SP | Brain |
| SA218 | 2013 | *Oreochromis niloticus* | FARM #19 | ES | Brain |
| SA220 | 2013 | *Oreochromis niloticus* | FARM #19 | SP | Brain |
| SA245 | 2013 | *Oreochromis niloticus* | FARM #20 | MG | Brain |
| SA256 | 2013 | *Oreochromis niloticus* | FARM #21 | CE | Brain |
| SA289 | 2013 | *Oreochromis niloticus* | FARM #22 | CE | Brain |
| SA330 | 2014 | *Oreochromis niloticus* | FARM #22 | SP | Brain |
| SA333 | 2014 | *Oreochromis niloticus* | FARM #22 | GO | Brain |
| SA341 | 2014 | *Oreochromis niloticus* | FARM #22 | MG | Brain |
| SA343 | 2014 | *Oreochromis niloticus* | FARM #22 | MG | Brain |
| SA346 | 2014 | *Oreochromis niloticus* | FARM #22 | MG | Brain |
| SA374 | 2014 | *Oreochromis niloticus* | FARM #22 | SP | Brain |
| SA375 | 2015 | *Oreochromis niloticus* | FARM #22 | SP | Brain |
| SA623 | 2015 | *Oreochromis niloticus* | FARM #23 | MG | Brain |
| SA627 | 2015 | *Oreochromis niloticus* | FARM #24 | MG | Brain |
| SA665 | 2015 | *Oreochromis niloticus* | FARM #24 | MG | Brain |
| SA719 | 2015 | *Oreochromis niloticus* | FARM #25 | MG | Brain |
| SA796 | 2015 | *Oreochromis niloticus* | FARM #26 | GO | Kidney |
| SA808 | 2016 | *Oreochromis niloticus* | FARM #27 | MG | Brain |
| SA887 | 2016 | *Oreochromis niloticus* | FARM #28 | MG | Kidney |
| SA929 | 2016 | *Oreochromis niloticus* | FARM #28 | MG | Brain |
| SA941 | 2016 | *Oreochromis niloticus* | FARM #29 | MG | Brain |
| SA959 | 2016 | *Oreochromis niloticus* | FARM #30 | MG | Kidney |
| SA972 | 2007 | *Oreochromis niloticus* | FARM #31 | MG | Brain |
| SD054 | 2007 | *Oreochromis niloticus* | FARM #32 | CE | Brain |
| SD056 | 2007 | *Oreochromis niloticus* | FARM #33 | CE | Brain |
| SD061 | 2007 | *Oreochromis niloticus* | FARM #34 | CE | Abscess |
| SD064 | 2007 | *Oreochromis niloticus* | FARM #35 | CE | Brain |
| SD068 | 2010 | *Oreochromis niloticus* | FARM #35 | CE | Kidney |
| SD092 | 2011 | *Oreochromis niloticus* | FARM #35 | AL | Kidney |
| SD120 | 2011 | *Oreochromis niloticus* | FARM #36 | CE | Kidney |
| SD137 | 2011 | *Oreochromis niloticus* | FARM #36 | CE | Kidney |
| SD140 | 2011 | *Oreochromis niloticus* | FARM #37 | CE | Kidney |
| SD142 | 2011 | *Oreochromis niloticus* | FARM #37 | CE | Kidney |
| SD143 | 2011 | *Oreochromis niloticus* | FARM #38 | CE | Kidney |
| SD145 | 2013 | *Oreochromis niloticus* | FARM #39 | CE | Kidney |
| SD280 | 2013 | *Oreochromis niloticus* | FARM #39 | CE | Abscess |
| SD281 | 2013 | *Oreochromis niloticus* | FARM #39 | CE | Abscess |
| SD282 | 2013 | *Oreochromis niloticus* | FARM #39 | CE | Abscess |
| SD283 | 2013 | *Oreochromis niloticus* | FARM #39 | CE | Abscess |
| SD284 | 2013 | *Oreochromis niloticus* | FARM #40 | CE | Kidney |
| SD285 | 2013 | *Oreochromis niloticus* | FARM #41 | CE | Kidney |
| SD286 | 2013 | *Oreochromis niloticus* | FARM #42 | CE | Brain |
| SD287 | 2014 | *Oreochromis niloticus* | FARM #43 | CE | Kidney |
| SD367 | 2014 | *Oreochromis niloticus* | FARM #43 | CE | Brain |
| SD370 | 2014 | *Oreochromis niloticus* | FARM #43 | CE | Brain |
| SD372 | 2006 | *Oreochromis niloticus* | FARM #43 | CE | Brain |
| SI022 | 2006 | *Oreochromis niloticus* | FARM #43 | PR | Brain |
| SI023 | 2006 | *Oreochromis niloticus* | FARM #43 | PR | Kidney |
| SI024 | 2006 | *Oreochromis niloticus* | FARM #43 | PR | Kidney |
| SI025 | 2006 | *Oreochromis niloticus* | FARM #43 | PR | Kidney |
| SI027 | 2006 | *Oreochromis niloticus* | FARM #44 | PR | Kidney |
| SI028 | 2006 | *Oreochromis niloticus* | FARM #44 | PR | Kidney |
| SI029 | 2014 | *Oreochromis niloticus* | FARM #44 | PR | Brain |
| SI444 | 2014 | *Oreochromis niloticus* | FARM #44 | PR | Brain |
| SI503 | 2015 | *Pseudoplatystoma corruscans* x *P. fasciatum* | FARM #44 | SP | Brain |
| SI674 | 2015 | *Oreochromis niloticus* | FARM #44 | MG | Brain |
| SI677 | 2015 | *Oreochromis niloticus* | FARM #44 | MG | Kidney |
| SI692 | 2015 | *Oreochromis niloticus* | FARM #44 | MG | Kidney |
| SI696 | 2015 | *Oreochromis niloticus* | FARM #44 | MG | Brain |
| SI698 | 2015 | *Oreochromis niloticus* | FARM #44 | MG | Kidney |
| SI699 | 2015 | *Oreochromis niloticus* | FARM #44 | MG | Brain |
| SI700 | 2015 | *Oreochromis niloticus* | FARM #44 | MG | Brain |
| SI701 | 2015 | *Oreochromis niloticus* | FARM #44 | MG | Brain |
| SI702 | 2015 | *Oreochromis niloticus* | FARM #45 | MG | Brain |
| SI705 | 2015 | *Oreochromis niloticus* | FARM #46 | MG | Brain |
| SI706 | 2015 | *Oreochromis niloticus* | FARM #46 | MG | Brain |
| SI711 | 2015 | *Oreochromis niloticus* | FARM #46 | MG | Kidney |
| SI712 | 2015 | *Oreochromis niloticus* | FARM #46 | MG | Kidney |
| SI713 | 2015 | *Oreochromis niloticus* | FARM #47 | MG | Kidney |
| SI714 | 2015 | *Oreochromis niloticus* | FARM #48 | MG | Brain |
| SI715 | 2015 | *Oreochromis niloticus* | FARM #48 | MG | Kidney |
| SI717 | 2015 | *Oreochromis niloticus* | FARM #48 | MG | Kidney |
| SI718 | 2015 | *Oreochromis niloticus* | FARM #49 | MG | Brain |
| SI720 | 2015 | *Oreochromis niloticus* | FARM #49 | MG | Kidney |
| SI790 | 2015 | *Oreochromis niloticus* | FARM #49 | MG | Brain |
| SI791 | 2015 | *Oreochromis niloticus* | FARM #49 | MG | Kidney |
| SI792 | 2015 | *Oreochromis niloticus* | FARM #49 | MG | Brain |
| SI797 | 2015 | *Oreochromis niloticus* | FARM #50 | MG | Kidney |
| SI798 | 2015 | *Oreochromis niloticus* | FARM #50 | MG | Kidney |
| SI819 | 2015 | *Oreochromis niloticus* | FARM #51 | MG | Kidney |
| SI826 | 2015 | *Oreochromis niloticus* | FARM #52 | MG | Kidney |
| SI831 | 2015 | *Oreochromis niloticus* | FARM #52 | MG | Kidney |
| SI839 | 2015 | *Oreochromis niloticus* | FARM #52 | MG | Kidney |
| SI841 | 2015 | *Oreochromis niloticus* | FARM #52 | MG | Kidney |
| SI842 | 2015 | *Oreochromis niloticus* | FARM #52 | MG | Kidney |
| SI852 | 2015 | *Oreochromis niloticus* | FARM #52 | MG | Kidney |
| SI870 | 2015 | *Oreochromis niloticus* | FARM #52 | MG | Kidney |
| SI875 | 2015 | *Oreochromis niloticus* | FARM #52 | MG | Kidney |
| SI876 | 2015 | *Oreochromis niloticus* | FARM #52 | MG | Kidney |
| SI913 | 2016 | *Oreochromis niloticus* | FARM #52 | MG | Brain |
| SI928 | 2016 | *Oreochromis niloticus* | FARM #52 | MG | Brain |
| SI954 | 2016 | *Oreochromis niloticus* | FARM #53 | MG | Kidney |
| SI970 | 2016 | *Oreochromis niloticus* | FARM #53 | MG | Brain |
