# Supplementary material for: Use of MALDI-TOF Mass Spectrometry for the Fast Identification of Gram-Positive Fish Pathogens
Source: Front Microbiol. 2017 Aug 9;8:1492. doi: 10.3389/fmicb.2017.01492 (PMC5552964; doi:10.3389/fmicb.2017.01492)
Supplement: Supplementary file 2 [file Table2.DOCX]

**Table S2. MALDI Biotyper result after inclusion of custom MSP of SDD SD64, SD92 and SD142 strains**

| **Strain** | **Best match** | | **Second best match** | | **Third best match** | |
| --- | --- | --- | --- | --- | --- | --- |
|  | **Organism** | **Score Value** | **Organism** | **Score Value** | **Organism** | **Score Value** |
| SD054 | *Streptococcus* *dysgalactiae* subsp. *dysgalactiae* SD64 | 2.497 | *Streptococcus* *dysgalactiae* subsp. *dysgalactiae* SD142 | 2.470 | *Streptococcus* *dysgalactiae* subsp. *dysgalactiae* SD92 | 2.352 |
| SD056 | *Streptococcus* *dysgalactiae* subsp. *dysgalactiae* SD64 | 2.480 | *Streptococcus* *dysgalactiae* subsp. *dysgalactiae* SD142 | 2.417 | *Streptococcus* *dysgalactiae* subsp. *dysgalactiae* SD92 | 2.204 |
| SD061 | *Streptococcus* *dysgalactiae* subsp. *dysgalactiae* SD64 | 2.438 | *Streptococcus* *dysgalactiae* subsp. *dysgalactiae* SD142 | 2.435 | *Streptococcus* *dysgalactiae* subsp. *dysgalactiae* SD92 | 2.410 |
| SD064 | *Streptococcus* *dysgalactiae* subsp. *dysgalactiae* SD64 | 2.458 | *Streptococcus* *dysgalactiae* subsp. *dysgalactiae* SD142 | 2.405 | *Streptococcus* *dysgalactiae* subsp. *dysgalactiae* SD92 | 2.291 |
| SD068 | *Streptococcus* *dysgalactiae* subsp. *dysgalactiae* SD142 | 2.497 | *Streptococcus* *dysgalactiae* subsp. *dysgalactiae* SD64 | 2.435 | *Streptococcus* *dysgalactiae* subsp. *dysgalactiae* SD92 | 2.242 |
| SD092 | *Streptococcus* *dysgalactiae* subsp. *dysgalactiae* SD64 | 2.320 | *Streptococcus* *dysgalactiae* subsp. *dysgalactiae* SD142 | 2.269 | *Streptococcus* *dysgalactiae* subsp. *dysgalactiae* SD92 | 2.237 |
| SD120 | *Streptococcus* *dysgalactiae* subsp. *dysgalactiae* SD64 | 2.346 | *Streptococcus* *dysgalactiae* subsp. *dysgalactiae* SD142 | 2.336 | *Streptococcus* *dysgalactiae* subsp. *dysgalactiae* SD92 | 2.193 |
| SD137 | *Streptococcus* *dysgalactiae* subsp. *dysgalactiae* SD142 | 2.338 | *Streptococcus* *dysgalactiae* subsp. *dysgalactiae* SD64 | 2.259 | *Streptococcus* *dysgalactiae* subsp. *dysgalactiae* SD92 | 2.255 |
| SD140 | *Streptococcus* *dysgalactiae* subsp. *dysgalactiae* SD142 | 2.531 | *Streptococcus* *dysgalactiae* subsp. *dysgalactiae* SD64 | 2.526 | *Streptococcus* *dysgalactiae* subsp. *dysgalactiae* SD92 | 2.248 |
| SD142 | *Streptococcus* *dysgalactiae* subsp. *dysgalactiae* SD142 | 2.384 | *Streptococcus* *dysgalactiae* subsp. *dysgalactiae* SD92 | 2.307 | *Streptococcus* *dysgalactiae* subsp *dysgalactiae* SD64 | 2.212 |
| SD143 | *Streptococcus* *dysgalactiae* subsp. *dysgalactiae* SD64 | 2.479 | *Streptococcus* *dysgalactiae* subsp. *dysgalactiae* SD142 | 2.476 | *Streptococcus* *dysgalactiae* subsp. *dysgalactiae* SD92 | 2.269 |
| SD145 | *Streptococcus* *dysgalactiae* subsp. *dysgalactiae* SD142 | 2.548 | *Streptococcus* *dysgalactiae* subsp. *dysgalactiae* SD64 | 2.484 | *Streptococcus* *dysgalactiae* subsp. *dysgalactiae* SD92 | 2.304 |
| SD280 | *Streptococcus* *dysgalactiae* subsp. *dysgalactiae* SD142 | 2.471 | *Streptococcus* *dysgalactiae* subsp. *dysgalactiae* SD64 | 2.413 | *Streptococcus* *dysgalactiae* subsp. *dysgalactiae* SD92 | 2.315 |
| SD281 | *Streptococcus* *dysgalactiae* subsp. *dysgalactiae* SD64 | 2.277 | *Streptococcus* *dysgalactiae* subsp. *dysgalactiae* SD142 | 2.224 | *Streptococcus* *dysgalactiae* subsp. *dysgalactiae* SD92 | 2.169 |
| SD282 | *Streptococcus* *dysgalactiae* subsp. *dysgalactiae* SD142 | 2.511 | *Streptococcus* *dysgalactiae* subsp. *dysgalactiae* SD64 | 2.484 | *Streptococcus* *dysgalactiae* subsp. *dysgalactiae* SD92 | 2.314 |
| SD283 | *Streptococcus* *dysgalactiae* subsp. *dysgalactiae* SD142 | 2.513 | *Streptococcus* *dysgalactiae* subsp. *dysgalactiae* SD64 | 2.509 | *Streptococcus* *dysgalactiae* subsp. *dysgalactiae* SD92 | 2.268 |
| SD284 | *Streptococcus* *dysgalactiae* subsp. *dysgalactiae* SD142 | 2.302 | *Streptococcus* *dysgalactiae* subsp. *dysgalactiae* SD64 | 2.258 | *Streptococcus* *dysgalactiae* subsp. *dysgalactiae* SD92 | 2.171 |
| SD285 | *Streptococcus* *dysgalactiae* subsp. *dysgalactiae* SD142 | 2.523 | *Streptococcus* *dysgalactiae* subsp. *dysgalactiae* SD64 | 2.449 | *Streptococcus* *dysgalactiae* subsp. *dysgalactiae* SD92 | 2.300 |
| SD286 | *Streptococcus* *dysgalactiae* subsp. *dysgalactiae* SD142 | 2.461 | *Streptococcus* *dysgalactiae* subsp. *dysgalactiae* SD64 | 2.405 | *Streptococcus* *dysgalactiae* subsp. *dysgalactiae* SD92 | 2.345 |
| SD287 | *Streptococcus* *dysgalactiae* subsp. *dysgalactiae* SD142 | 2.579 | *Streptococcus* *dysgalactiae* subsp. *dysgalactiae* SD64 | 2.549 | *Streptococcus* *dysgalactiae* subsp. *dysgalactiae* SD92 | 2.315 |
| SD367 | *Streptococcus* *dysgalactiae* subsp. *dysgalactiae* SD142 | 2.432 | *Streptococcus* *dysgalactiae* subsp. *dysgalactiae* SD64 | 2.383 | *Streptococcus* *dysgalactiae* subsp. *dysgalactiae* SD92 | 2.353 |
| SD370 | *Streptococcus* *dysgalactiae* subsp. *dysgalactiae* SD142 | 2.565 | *Streptococcus* *dysgalactiae* subsp. *dysgalactiae* SD64 | 2.432 | *Streptococcus* *dysgalactiae* subsp. *dysgalactiae* SD92 | 2.257 |
| SD372 | *Streptococcus* *dysgalactiae* subsp. *dysgalactiae* SD64 | 2.366 | *Streptococcus* *dysgalactiae* subsp. *dysgalactiae* SD142 | 2.351 | *Streptococcus* *dysgalactiae* subsp. *dysgalactiae* SD92 | 2.250 |
